# Online tool for adolescents' self-control practice: a pilot study

**DOI:** 10.25122/jml-2025-0115

**Published:** 2025-09

**Authors:** Alina Mihaela Munteanu, Teodor-Cristian Radoi, Adina Baciu, Cristiana Susana Glavce, Suzana Turcu

**Affiliations:** 1 Medical Anthropology, Francisc I Rainer Institute of Anthropology, Bucharest, Romania; 2 Politehnica University, Bucharest, Romania

**Keywords:** artificial intelligence (AI), self-control, teenagers, drama, Barratt impulsiveness scale (BIS)

## Abstract

Self-control is essential for youth navigating today’s technological and lifestyle challenges. Artificial intelligence (AI) offers scalable, personalized approaches to improve self-regulation through medical and educational interventions. This pilot research was conducted over a period of five months and structured into two studies, each comprising four phases. The first study included 180 adolescents, divided into two equal samples, to examine whether impulsivity varies according to high school profile. One group was drawn from Grigore Moisil Informatics College (theoretical high school), and the other from the National College of Arts Dinu Lipatti (vocational arts high school). Impulsivity was initially assessed using the Barratt Impulsiveness Scale (BIS). Drama students showed significantly higher levels of impulsivity, guiding the development of a targeted AI-driven (neural network-based) self-control intervention. The second study focused on the drama student cohort. Over the course of three months, the group participated in an online, AI-guided self-control education program. BIS was re-administered at the end of the intervention to measure changes. Pre-intervention data confirmed significantly higher impulsivity among drama students. Following the AI-based intervention, the group showed statistically significant improvements in self-control. The findings support the effectiveness of AI tools in fostering self-regulatory skills among adolescents and highlight their potential in health education and mental wellness, especially for anxiety, behavioral issues, and mild depression. The AI-guided, game-based cognitive training significantly reduced impulsivity in drama students, demonstrating its promise as a tool for improving adolescent self-control and psychological resilience.

## Introduction

Adolescence represents a critical stage in ontogeny, marking the transition to adulthood. This period is characterized by significant neurodevelopmental changes, often manifesting as reduced self-control and a tendency toward intensified emotional behavior [1]. Self-control, conceptualized as the ability to inhibit or delay automatic, impulsive reactions in favor of deliberative, goal-directed responses, is essential for adaptive social functioning and emotional resilience [2].

During adolescence, the brain undergoes asynchronous maturation. The prefrontal cortex, responsible for executive functions such as decision-making, planning, and impulse control, continues to develop and does not reach full maturity until approximately age 25 [3]. In contrast, the limbic system, associated with emotional reactivity and reward processing, matures earlier [4]. This developmental imbalance results in a heightened susceptibility to immediate gratification and emotional stimuli, as the prefrontal cortex is not yet fully capable of regulating impulses triggered by the limbic system.

This neurodevelopmental dynamic supports the dual-systems model of self-control [5], which emphasizes the coexistence of two neural systems: the 'hot' system, governed by the limbic structures and responsible for emotionally driven responses, and the 'cold' system, regulated by the prefrontal cortex and essential for reflective control. Effective self-control emerges from the dynamic interaction and balance between these systems.

Due to the dominance of the 'hot' system during adolescence, emotionally salient stimuli have a disproportionate influence on behavior [6]. Consequently, adolescents benefit from accessible, structured interventions that strengthen self-regulatory capacities. Artificial intelligence (AI) represents a promising area for developing such interventions, enabling the creation of personalized, adaptive educational programs that aim to optimize self-control and emotional regulation. Some institutions have already pioneered this approach: Stanford Medical University (2025) investigates the use of multimodal artificial intelligence models—integrating audio, video, and text data—to enable objective, scalable screening of depression and anxiety, aiming to enhance early detection through clinically relevant behavioral and digital markers [7]; the researchers at Carnegie Mellon University (2025) developed a virtual AI assistant aimed at supporting stress and emotion regulation [8].

Adolescents enrolled in dramatic arts education often exhibit personality traits that align with their vocational orientation, including spontaneity, creativity, heightened emotional sensitivity, physical expressiveness, and a significant focus on present experiences [9]. While these traits are advantageous in artistic contexts, they may also, when coupled with neurocognitive immaturity, increase vulnerability to impulsive and emotionally driven behavior [10].

Despite the value of spontaneity and emotional expressiveness in dramatic performance, achieving professional artistic competence requires emotional discipline, behavioral regulation in unpredictable situations, and the ability to distinguish personal affect from character representation [11]. These demands highlight the need for structured self-control training specifically adapted to adolescents performing arts education. However, to date, there is a lack of targeted digital interventions or training programs addressing impulse control and emotional regulation within this specific category of adolescents.

Accordingly, this study investigates the effectiveness of an AI-managed self-control education platform for adolescents enrolled in dramatic arts programs within the compulsory education curriculum of a public high school in Bucharest, Romania.

## Material and Methods

The study sample consisted of 180 adolescents aged between 14 and 17 years, with an equal gender distribution (1:1 ratio). Of these, 90 participants were first-year high school students enrolled in the dramatic arts program at the National College of Arts Dinu Lipatti, while the remaining 90 were students at the Grigore Moisil Informatics College, both located in Bucharest, Romania. Participant selection was based on voluntary enrollment.

Due to COVID-19 restrictions, the study was conducted entirely online via an online platform integrating cognitive training, self-monitoring, and assessment tools. This design enabled remote delivery of adaptive tasks and real-time data collection on impulsivity. Each participant created a personal account, anonymously coded (ID 1 to 180), through which they accessed the study materials and completed all tasks. The platform included the Barratt Impulsiveness Scale (BIS) [12], the Big Five Personality Test [13], and five cognitive games designed for self-control training. Informed consent was obtained from parents or legal guardians via an online form, ensuring compliance with pandemic-related health regulations.

The Barratt Impulsiveness Scale, a self-report measure initially developed by Ernest Barratt in 1965 and subsequently revised, is widely used in psychiatric and psychological assessments of impulsivity [12]. The scale comprises 30 items (11 of which are reverse-scored), organized into two scoring structures: a six-factor model (attention, motor, self-control, cognitive complexity, perseverance, and cognitive instability) and a three-factor model (attentional, motor, and non-planning impulsivity). The first adolescent adaptation of the BIS was conducted by Fossati, who culturally modified the items for use in Italian adolescent populations [14].

The Big Five Personality Test, which evaluates the traits of openness to experience, conscientiousness, extraversion, agreeableness, and neuroticism [13], was integrated into the platform to support the calibration of the AI system. This decision was informed by prior research indicating significant correlations between components of the BIS and the Big Five. In 1995, Patton, Stanford, and Barratt found associations between motor impulsivity and the traits of extraversion and conscientiousness[15]; Whiteside and Lynam observed relevant relationships between neuroticism and conscientiousness with motor and non-planning impulsivity [16]; and Miller and Lynam identified similar correlations across neuroticism, extraversion, and conscientiousness [17].

The AI network uploaded on the platform was trained on large datasets comprising BIS and Big Five item responses. Through iterative machine learning processes, the system identified patterns in impulsivity profiles and personality traits to generate personalized self-control training plans. These interventions were delivered through five cognitive games: anti-saccade [18], sequences [19], Go/no-go [20], Stroop [21], and puzzle games [22], all with adaptive difficulty levels [23]. Anti-saccade tasks require adolescents to suppress a reflexive eye movement toward a visual stimulus and instead look in the opposite direction, assessing their inhibitory control and attention regulation. Sequence tasks involve remembering and reproducing a specific order of items or actions, measuring working memory and cognitive flexibility. Stroop tasks ask adolescents to name the color of a word’s font while ignoring the word’s meaning (e.g., the word “red” printed in blue); they aim to improve selective attention and control over cognitive interference. Go/no-go tasks require adolescents to respond to certain stimuli (“go”) and inhibit their response to others (“no-go”), evaluating impulse control and response inhibition. Puzzle tasks involve solving logic or spatial problems and measure problem-solving ability, planning, and sustained attention. The cognitive tasks were selected based on prior research demonstrating their relevance in assessing and training components of self-control and executive functioning [18-22]. The combination of these tasks provides a comprehensive framework for developing and reinforcing self-regulatory skills within the context of a digital self-control training platform. The AI also selected cognitive-behavioral techniques tailored to the type of undesirable habit each participant actively sought to change, thereby supporting individualized habit-reversal strategies.

In addition to assessment tools and games, each participant's account featured a virtual space for self-monitoring progress during the transfer phase and for storing personal results. Prior to the research, the platform was internally pilot-tested with a small sample of 15-year-old volunteer students (*n* = 30) to evaluate usability, interface clarity, and item response coherence. Minor adjustments were made to the phrasing of instructions and feedback loops based on pilot feedback. Additionally, the initial inter-group assessment between theoretical and vocational students acted as a quasi-experimental pre-pilot phase to refine content targeting and adaptive parameters. The study spanned 5 months, from October 2021 to March 2022, and was structured into four phases: pretest, training, transfer, and feedback.

**Figure 1:**
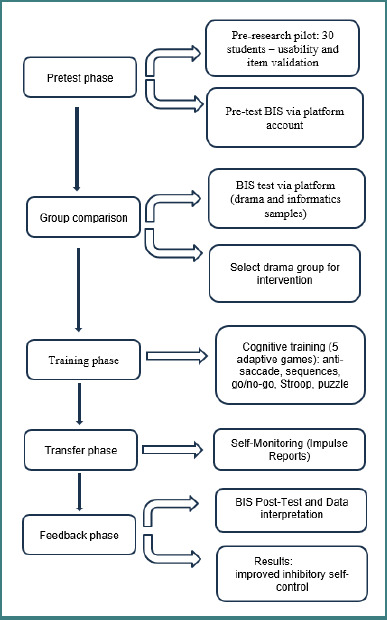
AI-based self-control intervention

During the pretest phase, participants completed the BIS in their online accounts. In the training phase, the AI system (neural network) used these assessments to assign daily personalized tasks, including adaptive difficulty games. The training two-month phase consisted of tailored self-control exercises based on BIS scores, as recommended by the AI system. During the transfer phase, the platform prompted students to record behavioral responses to specific impulses (choosing not to respond immediately to a temptation; practicing breathing techniques before reacting impulsively; delaying desired actions, such as eating a snack or checking the social media applications; practicing distraction strategies) in real-world contexts, reinforcing the delay strategies practiced in training. The feedback phase included a reassessment of impulsivity using the same instrument, BIS, and provided comparative visualizations of pre- and post-intervention data. Initial and final scores were stored and statistically analysed using the Shapiro-Wilk test for normality, the Wilcoxon signed-rank test for paired data, and effect size calculations to assess the magnitude of score changes. These analyses were conducted to evaluate the effectiveness of the intervention. Figure 1 illustrates the workflow of the AI-based self-control intervention applied in this study.

## Results

### Descriptive statistics

To ensure comparability between groups, participants were matched based on three key criteria: study domain (drama vs. informatics), age range (14–17 years, with a concentration around 15–16), and gender distribution (1:1 ratio). Each group included 90 students, with approximately equal numbers of male and female participants. Age distributions were analyzed to confirm equivalence in developmental stage, and group composition was controlled to reflect a similar level of academic settings (urban high schools, all students were in the first year). The gender distribution was balanced across the groups: the drama group consisted of 45 girls (50%) and 45 boys (50%), while the informatics group comprised 46 girls (51.1%) and 44 boys (48.9%). Age distributions were also comparable, with both groups predominantly composed of 15 and 16-year-olds. Specifically, 62.2% of students in the drama group and 64.4% in the informatics group were aged 15, while those aged 16 represented 30% and 32.2% respectively. Minimal variation was observed at the extremes (ages 14 and 17). In addition, participants were broadly similar in terms of family background and urban–rural distribution, thus supporting demographic equivalence between the groups. Moreover, the selected participants had no documented clinical diagnosis or evidence of psychiatric or psychological disorders at the time of the study.

### Comparative analysis of BIS scores between the two study groups

A comparative analysis of the BIS scores revealed notable differences between the two cohorts in the drama program and those in the informatics program:

The minimum score among informatics students was 63, while the drama group recorded a slightly lower minimum of 61, indicating that one student in the drama group demonstrated lower impulsivity than any student in the informatics group (Table 1).

**Table 1:** Comparative BIS scores

Weighted Average	Percentile						
	5	10	25	50	75	90	95
Barratt Scores – Drama	67.55	71.00	73.00	75.00	77.00	78.00	79.50
Barratt Scores – Informatics	64.59	65.92	66.95	68.75	70.67	72.52	73.21

The highest BIS score recorded in the informatics group was 75, whereas in the drama group, it reached a maximum of 83, indicating a greater level of impulsivity among drama students. Median scores further support this pattern, with informatics students scoring 68.75 compared to 75.00 in the drama group, suggesting overall higher impulsivity in the latter. Notably, the 90^th^ percentile score in the informatics group was 72.52—equivalent to the 25^th^ percentile in the drama group— and highlighted a relevant difference in score distribution, thus reinforcing the trend of high impulsivity among drama students.

To determine the statistical significance of the observed differences, a Mann–Whitney U test was conducted to compare the mean ranks between the two groups. The analysis revealed a statistically significant disparity in impulsivity scores, with the drama students showing a higher mean rank (128.36) compared to the informatics students (52.64), U = 642.500, Z = -9.782, *P* < .001. The effect size (r = -0.729) indicates a strong effect (r ≥ 0.5) [24]. These findings suggest a potential relationship between students’ academic discipline and their levels of impulsivity.

During the training phase, participants applied the AI-personalized programs on the digital platform. The six-week training sessions were scheduled for 15–20 minutes, four times per week. Each game was designed with a progressively increasing difficulty level: advancement to higher levels was triggered once a participant achieved a threshold of 60 correct responses within a one-minute interval (one correct task/one second). Performance scores were automatically recorded in each participant's personal account on a weekly basis.

The study evaluated participants’ performance across five categories of cognitive tasks previously mentioned: anti-saccade, sequence, Stroop, go/no-go, and puzzle tasks. Median performance scores were recorded during both the initial and final weeks of the intervention. To assess changes over time, the Wilcoxon signed-rank test was performed due to the non-parametric nature of the data. The results were interpreted within a psychological framework to assess the program's efficacy in enhancing self-regulation and cognitive control. A summary of the comparative data is presented in Table 2.

**Table 2:** Median scores for all tasks at the start and end of training

Task category	Week 1 median	Week 4 median	Z (Wilcoxon)	*P* value	Positive ranks
Anti-saccade	49.00	55.00	-8.260	*P* < .001	90
Sequence	48.00	58.00	-8.309	*P* < .001	90
Stroop	36.00	48.00	-8.357	*P* < .001	90
Go/No-Go	41.00	51.00	-8.314	*P* < .001	90
Puzzle	39.00	47.00	-8.258	*P* < .001	90

Table 3 presents the descriptive statistics of individual scores recorded for each task during the first and final weeks of training.

**Table 3:** Descriptive statistics of individual scores

Individual scores	Mean	Std. Deviation
Puzzle initial	39.84	3.499
Puzzle final	48.92	4.672
Sequence initial	50.76	4.781
Sequence final	61.27	4.656
Stroop initial	37.47	2.973
Stroop final	48.97	2.870
Go/no-go initial	42.72	3.519
Go/no-go final	53.16	4.116
Anti-saccade initial	41.97	5.299
Anti-saccade final	56.56	4.337

### Anti-saccade task

To evaluate the outcomes of the anti-saccade task, the Wilcoxon signed-rank test was performed, as the data distribution was non-normal (Shapiro-Wilk test: *W* = 0.834, *P* < .001). Statistical significance was established at the conventional threshold of *P* < 0.05, indicating meaningful associations between the study variables [24].

The analysis suggested a statistically significant difference between the median scores obtained in the first week of training (*M* = 40.00) and those recorded at the end of the program (*M* = 55.00). The Wilcoxon test yielded a *Z* score of –8.260, *P* < .001, indicating a substantial improvement in the number of correct responses by the end of the training period.

**Figure 2:**
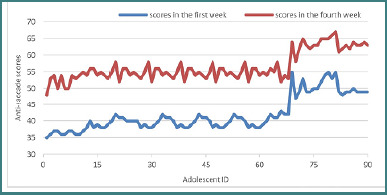
The evolution of scores on the anti-saccade tasks after four weeks of training

These findings suggest that the anti-saccade task was effective in enhancing inhibitory control among all adolescent participants. The progression of scores over the course of the intervention is illustrated in Figure 2.

### Sequence task

The sequence task, designed to assess working memory, pattern recognition, and inhibitory control, showed a statistically significant improvement in participant performance. Median scores increased from *M* = 48.00 in the first week to *M* = 58.00 in the final week of the intervention. Analysis using the Wilcoxon signed-rank test indicated a significant effect, with *Z* = –8.309, *P* < .001.

**Figure 3:**
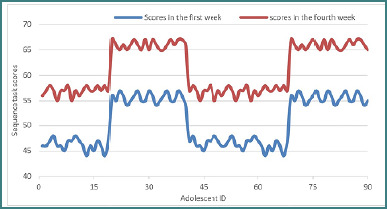
Sequence scores after four weeks of training

Notably, the number of positive ranks was 90, indicating that all participants demonstrated improved performance in this task by the end of the training period. These results suggest a meaningful enhancement of inhibitory control and cognitive processing as a result of the intervention. The progression of scores from the first to the fourth week is illustrated in Figure 3.

### Stroop task

Performance on the Stroop task, which evaluates cognitive flexibility, selective attention, and inhibitory control, showed a substantial improvement over the course of the intervention. The median score increased from *M* = 36.00 in the initial week to *M* = 48.00 by the final week. The Wilcoxon signed-rank test revealed a significant effect, with *Z* = –8.357, *P* < .001, indicating a highly significant increase in the number of correct responses per minute.

**Figure 4:**
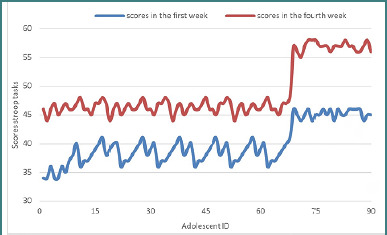
The evolution of scores – stroop tasks

All participants (*n* = 90) exhibited positive ranks, indicating a consistent improvement in executive functioning, particularly in the ability to resolve interference. The evolution of scores from the first week to the final week is depicted in Figure 4.

### Go/no-go task

Performance on the Go/no-go task, which targets response inhibition, demonstrated significant improvement. Median scores increased from *M* = 41.00 in the initial week to *M* = 51.00 in the final week. Statistical analysis using the Wilcoxon signed-rank test revealed a significant effect, with *Z* = –8.314, *P* < .001, indicating a notable enhancement in the final scores relative to the initial ones.

**Figure 5:**
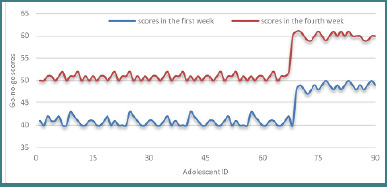
Go/No-Go scores after the training period

All 90 participants showed improved performance, further emphasizing the uniform benefit of the intervention in enhancing impulsivity control and response accuracy. The progression of scores is illustrated in Figure 5.

### Puzzle task

In the puzzle task, which assesses problem-solving, abstract reasoning, and executive control, participants demonstrated significant improvement in performance. The median scores increased from *M* = 39.00 to *M* = 47.00 during the study period. Statistical analysis using the Wilcoxon signed-rank test revealed this change to be statistically significant (*Z* = –8.258, *P* < .001).

**Figure 6:**
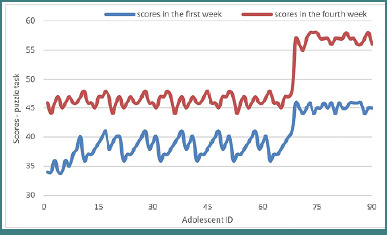
The evolution of scores of the puzzle-type tasks after training

As with the other tasks, all 90 participants exhibited positive rank changes, indicating that the intervention was effective in enhancing higher-order cognitive processing. The statistical results also indicated a relevant increase in the number of correct answers by the end of the program, further suggesting that the training was successful in improving executive control among the adolescents in the target group. The evolution of scores from the first week to the final week is presented in Figure 6.

Throughout the transfer phase, adolescents monitored and recorded the latency between the initial urge to engage in an undesired behavior and the moment the behavior was enacted. Within six weeks, participants applied cognitive-behavioral strategies aimed at increasing this latency, as reported in weekly self-reports. To assess changes over time, a Wilcoxon signed-rank test was conducted to compare median latency values from the first and final weeks. The analysis yielded a statistically significant increase in latency (*P* < .001), suggesting enhanced impulse inhibition and improved executive functioning.

## Discussion

The primary aim of this research was to investigate differences in impulsivity among adolescents based on their educational specialization and to evaluate the effectiveness of an AI-supported self-control education program. Initial findings revealed significantly higher levels of impulsivity among students attending a vocational arts high school compared to those from a theoretical informatics high school. These results are consistent with the existing literature, which suggests that impulsivity is a multifaceted personality trait influencing everyday behavior [25] and may also contribute to the selection of educational pathways during high school. From this perspective, the elevated impulsivity observed in arts students likely reflects greater disinhibition and emotional reactivity, underscoring the importance of targeted interventions such as the AI-based self-control program.

Additionally, these findings are consistent with previous research. For instance, Hall *et al*. conducted a study on the effectiveness of an emotional regulation and impulse control intervention (ERIC) for vulnerable youth, which demonstrated significant improvements in impulse and emotion management, underscoring the value of integrated approaches for supporting self-regulation [26]however the vast majority of psychological interventions are disorder specific. Novel psychological approaches that adequately acknowledge the psychosocial complexity and transdiagnostic needs of vulnerable young people are urgently needed. A modular skills-based program for emotion regulation and impulse control (ERIC. Similarly, Kip *et al*. examined the efficacy of a self-control training application, finding significant increases in self-control scores and reductions in aggression among students, which highlights the potential of mobile applications in self-regulation interventions [27].

In the feedback stage, we conducted a statistical analysis of the median initial and final scores on the BIS scale, which showed a decrease in the median score from *M* = 75.00 to *M* = 67.00. The Wilcoxon signed-rank test revealed a statistically significant difference between these scores (*Z* = –8.184, *P* < .001), indicating a meaningful reduction in impulsivity following the self-control training program. These results suggest that the intervention successfully contributed to a decrease in impulsivity among the adolescents in the target group. The findings are consistent with other pilot studies. For example, Rodríguez-Ruiz *et al*. examined the use of AI tools to support psychological traits such as self-control, self-esteem, and self-efficacy in university students, finding a significant correlation between AI tool use and improvements in self-control, further supporting the potential of AI-based technologies for fostering self-regulation in educational contexts [28].

These results are further supported by previous research. For example, brain mapping studies have identified distinct activity patterns in the dorsolateral prefrontal cortex (DLPFC) and ventromedial prefrontal cortex (vmPFC) among individuals exhibiting high levels of self-control [29]. In addition, a longitudinal study involving two adolescent cohorts found that engagement in structured arts-based activities, such as drama and dance, was associated with a reduced likelihood of antisocial or criminal behavior up to two years later [30]. Moreover, a 23-year longitudinal study demonstrated that higher self-control during adolescence predicted more successful outcomes in adulthood, particularly in the domains of romantic relationships and occupational functioning [31].

These findings contribute to the growing body of literature that demonstrates self-control is not a fixed trait, but rather one that can be optimized through targeted interventions. This study uniquely combines educational specialization differences with the evaluation of an AI-supported self-control training program, addressing impulsivity in adolescents from both vocational arts and theoretical informatics backgrounds. The success of the AI-supported training program highlights the value of personalized, data-informed approaches in adolescent self-regulation. Moreover, the integration of drama-based educational components, beyond the cognitive training tasks, may provide additional benefits by engaging emotional and expressive faculties, which are particularly relevant in adolescence [32]. Considering the relatively early stage of research in this interdisciplinary area, further studies are needed to explore long-term outcomes and scalability. Nonetheless, the results suggest that combining arts education with AI-driven interventions can provide a complementary and efficient strategy for enhancing emotional and behavioral control in youth.

### Study limitations

While the findings offer positive insights into the potential of AI-supported cognitive training for reducing impulsivity in adolescents, several limitations should be noted. First, the absence of a control group limits the ability to fully distinguish the specific effects of the intervention from natural developmental changes or external influences.

Second, although group equivalence was carefully monitored, the lack of random assignment may have introduced selection bias, particularly in relation to individual differences such as motivation or educational orientation. Additionally, the exclusive reliance on self-report measures for assessing impulsivity may pose a risk of subjective bias, despite the practical advantages of such instruments in a remote, pandemic-affected context.

Lastly, while the platform automatically recorded performance metrics, qualitative aspects such as user engagement and motivational dynamics were not explored, representing a potentially valuable direction for future research.

## Conclusion

This article examines the effectiveness of an AI-managed platform in promoting the development of self-control among adolescents studying dramatic arts. Based on the initial self-reported impulsivity assessment using the BIS scale, the cognitive training program demonstrated significant improvements in self-control over a period of approximately four months. These improvements were subsequently applied during the transfer stage, where adolescents utilized cognitive techniques to delay undesirable habits. A notable increase in the latency time between the onset of impulses and the execution of these habits was observed after six weeks of implementing the techniques and engaging in self-monitoring.

A comparative analysis of the initial and final BIS scores revealed a significant reduction in impulsivity, further supporting the program's effectiveness for the sample of adolescents. The results highlight the potential of AI-assisted educational programs in fostering self-control among this specific cohort. Moreover, the findings suggest that such methods are not only effective but also accessible and user-friendly for managing emotions and behaviors across various life situations, including both personal and artistic contexts.

For future research, two main directions are proposed. The first is cross-sectional, aimed at adapting the program for a broader adolescent population, irrespective of their academic or artistic background. This phase will also explore the correlations between self-control development and artistic growth. The second direction is longitudinal, which will extend the evaluation of the program's impact on academic performance and social adaptation. Additionally, this approach will assess the long-term effectiveness of the methods and techniques employed by the AI-managed platform in promoting sustained self-control development.

## References

[1] Padmanabhan A, Geier CF, Ordaz SJ, Teslovich T, Luna B (2011). Developmental changes in brain function underlying the influence of reward processing on inhibitory control. Dev Cogn Neurosci.

[2] Baumeister RF, Vohs KD, Tice DM (2007). The strength model of self-control. Current Directions in Psychological Science.

[3] Arain M, Haque M, Johal L, Mathur P, Nel W, Rais A (2013). Maturation of the adolescent brain. Neuropsychiatr Dis Treat.

[4] Steinberg L (2008). A Social Neuroscience Perspective on Adolescent Risk-Taking. Dev Rev.

[5] Casey BJ, Getz S, Galvan A (2008). The adolescent brain. Dev Rev.

[6] Metcalfe J, Mischel W (1999). A hot/cool-system analysis of delay of gratification: dynamics of willpower. Psychol Rev.

[7] Stanford Partnership in AI-Assisted Care Behavioral health. Stanford Medicine.

[8] Fang A, Chhabria H, Maram A, Zhu H (2024). Practicing stress relief for the everyday: designing social simulation using VR, AR, and LLMs. arXiv.

[9] Wirag A (2024). Why drama has an impact on student personality development: a qualitative interview study with EFL drama students. NJ: Drama Australia Journal.

[10] Green R, Meredith LR, Mewton L, Squeglia LM (2023). Adolescent Neurodevelopment Within the Context of Impulsivity and Substance Use. Curr Addict Rep.

[11] Mehler M, Balint E, Gralla M, Pößnecker T, Gast M, Hölzer M (2024). Training emotional competencies at the workplace: a systematic review and metaanalysis. BMC Psychol.

[12] BARRATT ES (1965). FACTOR ANALYSIS OF SOME PSYCHOMETRIC MEASURES OF IMPULSIVENESS AND ANXIETY. Psychol Rep.

[13] McCrae RR, Costa PT, John OP, Robins RW, Pervin LA (1999). A five-factor theory of personality. Handbook of personality: Theory and research.

[14] Fossati A, Barratt ES, Acquarini E, Di Ceglie A (2002). Psychometric properties of an adolescent version of the Barratt Impulsiveness Scale-11 for a sample of Italian high school students. Percept Mot Skills.

[15] Patton JH, Stanford MS, Barratt ES (1995). Factor structure of the Barratt impulsiveness scale. J Clin Psychol.

[16] Whiteside SP, Lynam DR (2001). The Five Factor Model and impulsivity: using a structural model of personality to understand impulsivity. Pers Individ Dif.

[17] Miller JD, Flory K, Lynam DR, Leukefeld C (2003). A test of the four-factor model of impulsivity-related traits. Pers Individ Dif.

[18] Magnusdottir BB, Faiola E, Harms C, Sigurdsson E, Ettinger U, Haraldsson HM (2019). Cognitive Measures and Performance on the Antisaccade Eye Movement Task. J Cogn.

[19] Baniqued PL, Allen CM, Kranz MB, Johnson K, Sipolins A, Dickens C (2015). Working Memory, Reasoning, and Task Switching Training: Transfer Effects, Limitations, and Great Expectations?. PLoS One.

[20] Rubia K, Smith AB, Woolley J, Nosarti C, Heyman I, Taylor E (2006). Progressive increase of frontostriatal brain activation from childhood to adulthood during event-related tasks of cognitive control. Hum Brain Mapp.

[21] Scarpina F, Tagini S (2017). The Stroop Color and Word Test. Front Psychol.

[22] Knoll LJ, Fuhrmann D, Sakhardande AL, Stamp F, Speekenbrink M, Blakemore SJ (2016). A Window of Opportunity for Cognitive Training in Adolescence. Psychol Sci.

[23] Munteanu AM, Rădoi TC, Glavce CS, Petrescu M, Turcu S, Borosanu A (2025). AI-based intervention to enhance self-control in adolescents studying drama—a pilot study. J Mind Med Sci.

[24] Field AP (2018). Discovering statistics using IBM SPSS Statistics.

[25] Sharma L, Markon KE, Clark LA (2014). Toward a theory of distinct types of "impulsive" behaviors: A meta-analysis of self-report and behavioral measures. Psychol Bull.

[26] Hall K, Youssef G, Simpson A, Sloan E, Graeme L, Perry N (2021). An Emotion Regulation and Impulse Control (ERIC) Intervention for Vulnerable Young People: A Multi-Sectoral Pilot Study. Front Psychol.

[27] Kip H, Da Silva MC, Bouman YHA, van Gemert-Pijnen LJEWC, Kelders SM (2021). A self-control training app to increase self-control and reduce aggression-A full factorial design. Internet Interv.

[28] Rodríguez-Ruiz J, Marín-López I, Espejo-Siles R (2025). Is artificial intelligence use related to self-control, self-esteem and self-efficacy among university students?. Educ Inf Technol.

[29] Hare TA, Camerer CF, Rangel A (2009). Self-control in decision-making involves modulation of the vmPFC valuation system. Science.

[30] Bone JK, Bu F, Fluharty ME, Paul E, Sonke JK, Fancourt D (2022). Arts and Cultural Engagement, Reportedly Antisocial or Criminalized Behaviors, and Potential Mediators in Two Longitudinal Cohorts of Adolescents. J Youth Adolesc.

[31] Allemand M, Job V, Mroczek DK (2019). Self-control development in adolescence predicts love and work in adulthood. J Pers Soc Psychol.

[32] Munteanu A, Petrescu M, Turcu S (2024). The importance of teaching self-control to adolescents in the age of artificial intelligence. Ann Roum Anthropol.

